# Microbial biomarkers as indicators of sperm viability in an insect

**DOI:** 10.1098/rsos.240734

**Published:** 2024-09-11

**Authors:** Kathryn B. McNamara, Ashley M. Dungan, Linda L. Blackall, Leigh W. Simmons

**Affiliations:** ^1^ School of BioSciences, University of Melbourne, Royal Parade, Parkville, Victoria 3010, Australia; ^2^ Centre for Evolutionary Biology & School of Biological Sciences, University of Western Australia, 35 Stirling Highway, Crawley 6009, Western Australia, Australia

**Keywords:** reproductive microbiome, sperm competition, sexual selection

## Abstract

Our understanding of microbial variation in male reproductive tissues is poorly understood, both regarding how it varies spatially across different tissues and its ability to affect male sperm and semen quality. To redress this gap, we explored the relationship between male sperm viability and male gut and reproductive tract microbiomes in the Pacific field cricket, *Teleogryllus oceanicus*. We selected cohorts of males within our populations with the highest and lowest natural sperm viability and characterized the bacterial microbiota present in the gut, testes, seminal vesicle, accessory glands and the spermatophore (ejaculate) using 16S ribosomal RNA gene metabarcoding. We identified bacterial taxa corresponding to sperm viability, highlighting for the first time an association between the host’s microbial communities and male competitive fertilization success. We also found significant spatial variation in bacterial community structure of reproductive tissue types. Our data demonstrate the importance of considering the microbial diversity of both the host gut and reproductive tract when investigating male fertility in wildlife and potentially human clinical settings.

## 1. Introduction

Internal tissues and organs are not sterile and instead contain populations of bacteria, fungi and viruses, collectively known as the microbiome. These resident communities function in both homeostatic symbiosis and pathogenic dysbiosis. To date, research has focused primarily on the microbiota of the gut, highlighting the inextricable link between microbial diversity and individual health [1–3]. More recently, research has broadened to consider the microbiomes of other organs and tissues, such as the reproductive tract [4,5]. The reproductive microbiome broadly encompasses the microbial communities associated with any reproductive structure (for example, reproductive tracts, gametes or intromittent or genital-clasping organs) or fluid (for example, ejaculates and vaginal secretions). Although there is growing evidence that reproductive microbiomes are diverse [4], there is currently a lack of understanding as to how this diversity impacts host mating dynamics and reproductive fitness. Thus far, studies have focused primarily on microbial variation in human (or human-model) female reproductive tracts in clinical studies, to address the mechanisms of infertility and pregnancy loss and complications [6]. However, our understanding of the relationship between male fertility and the microbiome (gut and reproductive tract) has only relatively recently been the focus of intensive research.

Recent evidence suggests that semen and male reproductive tissues contain a distinct microbiome [7–9]. Human studies demonstrate high variability in the host male’s microbial communities, which reside primarily in the lower genital tract [10]. Studies of human couples show that semen has a greater bacterial diversity, albeit lower bacterial count, than vaginal samples [11]. Studies of male reproductive microbiota typically examine the impact of experimental application of isolated bacteria *in vitro* [12], rather than considering how normal variation in naturally occurring reproductive microbes shape host fertility, *in vivo*. These studies demonstrate across vertebrate models that microbes are a biologically relevant cause of sperm dysfunction, and microbially induced reproductive dysfunction may impose significant fitness costs on individuals. These *in vitro* assays have demonstrated that bacteria can damage sperm via sperm agglutination and plasma membrane disruption [12–14] and DNA damage [15], which can lead to reduced fertilizing qualities, such as motility and viability [12,16–18], and correspondingly a negative effect on male reproductive fitness. While studies of experimental infection with individual taxa are informative, it is important to examine the microbiome as a whole, to unravel its biological relevance for male sperm quality and fertility, and to reveal the potential for targeted probiotic interventions to improve male fertility. One study of the human testicular microbiome has demonstrated that normozoospermic men have small amounts of bacteria in the testis, with Actinobacteria, Bacteroidota, Firmicutes and Proteobacteria as the dominant phyla [7]. There is also emerging evidence that the gut microbiome, and in particular gut dysbiosis, can shape male fertility. These studies have primarily focused on murine model systems as a means to understand human fertility [5,19,20]. Such studies highlight the potential for the gut microbiome-mediated ‘rescuing’ of fertility damaged through environmental stressors [20]. Thus, both gut and reproductive microbiomes should be considered in concert for their potential role in mediating male fertility. Indeed, microbe-ejaculate interactions are increasingly recognized as a potential driving force in post-copulatory sexual selection [4].

For many polyandrous species in which the sperm of multiple males compete for fertilization (sperm competition), relative paternity success is largely determined by the number of viable sperm and/or the fertilizing efficiency of those sperm [21–24]. The demonstrated capacity for microbes to alter sperm viability and key indices of sperm fertilizing potential, such as motility and longevity, means that host microbial infection or microbial variation can shape male sperm competitiveness and fitness. Currently, there is limited empirical investigation of microbial impacts on the outcome of sperm competition in insects, and these have primarily focused on the impact of individual bacterial endosymbiont species (*Wolbachia pipientis*) on male competitive fertilization success [25]. *Wolbachia* is a maternally inherited, intracellular bacterium that is thought to infect up to 75% of arthropod species [26]. *Wolbachia* is implicated in the manipulation of offspring sex ratios and/or host reproductive compatibilities to increase its own transmission [25]. Evidence for *Wolbachia’s* impact on sperm competitiveness, however, is equivocal [27,28].

Investigation of microbe-mediated fertility has typically focused on human and human-model mammalian species. For invertebrate literature, there is comparatively little research, and most have focused on the effects of experimental infections on sperm traits [29]. Iridovirus IIV−6/CrIV infection of the cricket, *Gryllus texensis*, resulted in little or no sperm motility within the ejaculate, rendering males infertile [30], while bacterial infection with *Serratia marcescens* reduced sperm viability in Pacific field crickets, *Teleogryllus oceanicus* [31]. In a growing number of invertebrate species [32,33], immune factors have been identified in the ejaculate at higher levels than in the circulating haemolymph of the male [32], suggesting that sperm-microbe interactions are likely important in the evolution of ejaculate components.

We used *T. oceanicus* (Le Guillou, Orthoptera: Gryllidae) as a model species to explore the relationship between male fertility and the microbial communities present in their gut, seminal vesicle, testes, accessory glands and ejaculate (spermatophore). *T. oceanicus* is highly polyandrous, and male sperm competitive ability (male proportional paternity success when competing with other males for access to a female’s ova) is determined by the proportion of live sperm present in the ejaculate—sperm viability [21], but not sperm number [34]. Male sperm viability is variable between males and is affected by the seminal proteins present in the ejaculate [35,36]. Moreover, there are negative phenotypic and genetic correlations between male sperm viability and anti-bacterial immunity [37]. Characterization of the seminal fluid proteome in *T. oceanicus* identified two proteins that may protect sperm from bacteria: a transferrin-like protein that is correlated with ejaculate quality in other species [33], and an immunologically active protein in crickets, apolipophorin-III [38]. While the gut microbiome of this species has also recently been characterized [39,40], its reproductive microbiome has never been assessed, nor has the direct relationship between gut or reproductive tract microbial diversity and fertility. Here, we sampled two cohorts of males, one with naturally high sperm viability and the other with naturally low sperm viability. We then analyzed the gut and reproductive tract microbial communities in these tissues. We had two predictions: first, there would be variation in the microbial communities between the gut and reproductive tract, and second, that this variation in male reproductive and/or gut microbial diversity would correlate with male sperm viability, which contributes to male fitness.

## 2. Material and methods

### 2.1. Experimental animals

Experimental animals were derived from a large, outbred laboratory stock population that was initiated from and annually supplemented with wild-caught individuals from Carnarvon, Western Australia. Individuals were housed in a constant temperature room at 25°C on a 12 h light: 12 h dark photoperiod. The population was checked daily and adult males were selected (on the day of adult eclosion) from this stock population and isolated individually in plastic containers (7 × 7 × 5 cm), supplied with ad libitum cat chow and a water feeder (a vial filled with water and plugged with cotton wool).

### 2.2. Spermatophore removal

When males were 13 days post adult eclosion, they were weighed, and the male’s genital pouch was inspected for the presence of a spermatophore. If present, the spermatophore was removed in a laminar flow hood with sterilized forceps and placed in a 1.5 ml microcentrifuge tube for later microbiota analysis. Males were returned to their containers for 24 h without food (to empty the gut of food prior to dissection). Males were then again inspected for a spermatophore, and if present, the spermatophore was removed and immediately assayed for sperm viability. Males that did not have a spermatophore present on day 13 had their sperm viability analysis conducted on day 14, and an additional spermatophore was removed on day 15 for microbiota analysis. Thus, all sperm viability assays were conducted on males of the same age. Males that did not have a second spermatophore present were discarded. Males were frozen at −80°C prior to dissection (see below).

### 2.3. Sperm viability analysis

Sperm viability was assessed using a Live:Dead Sperm Viability Kit (Molecular Probes, Eugene, OR, USA). The kit differentially dyes viable (green) and dead (red) sperm in the ejaculate, allowing an estimate of the proportion of sperm viable. Sperm viability in *T. oceanicus* is a predictor of male competitive fertilization success [21]. The spermatophore was severed at the neck and the sperm was allowed to disperse into 20 μl of Beadle saline (128.3 mM NaCl, 4.7 mM KCl, 23 mM CaCl_2_) on a glass slide. A 5 μl subsample of this solution was taken and gently mixed on a glass slide with 5 μl of a 1:50 dilution of 1 mM SYBR−14, and incubated in the dark for 10 min. Next, 2 μl of propidium iodide was mixed in, and the solution was incubated for a further 10 min. A cover slip was placed over the solution and the sperm counted using a fluorescent optical filter at 200 × magnification. The colour (viability) of the first 500 sperm observed, from several randomly selected fields on the slide, was determined. Approximately 10% of the sperm sampled can be both red and green stained (‘doubly-stained’) [41]; these were considered ‘dead’. Thus, the viability of the sample was calculated as the proportion of green sperm in the total number of sperm (green, red and doubly stained).

### 2.4. Gut and reproductive tract dissection

While all males had their viability assayed on day 14, two males had their spermatophore removed (and were killed) for microbiome analysis on day 15, rather than day 13 (*n* = one low- and one high-viability male). All frozen males were then dissected, and their gut and reproductive tract microbiota were characterized. Males were first thawed on ice (after approximately 2 weeks at −80°C). Males were then immersed in 70% ethanol for 5 mins, and then dissected under a laminar flow hood using sterilized dissecting equipment. For each male, the gut (including the mid- and hind-gut, removed posteriorly to the proventriculus), testes, seminal vesicle and accessory glands were removed and stored in separate 1.5 ml microcentrifuge tubes, and stored at −80°C. All equipment was sterilized between males using 70% ethanol. Gut and reproductive tract samples, in addition to the previously harvested spermatophores, were packed in liquid nitrogen and shipped overnight to The University of Melbourne for DNA extraction and sequencing.

### 2.5. DNA extraction and sequencing of bacterial 16s ribosomal RNA genes

Dissected organs, including spermatophores, were first homogenized in 300 μl of sterile phosphate-buffered saline (PBS; Sigma-Aldrich, Australia) in a microcentrifuge tube using a pestle. Approximately half of the sample was then removed and placed in a new 1.5 ml microcentrifuge tube for DNA extraction, and the remaining sample was stored at −80°C. DNA extractions were completed as previously described by Hartman *et al*. [42]. Blank extractions without sample homogenate (*n* = 7) were used to test for reagent contamination.

Extracted DNA was amplified by PCR in triplicate using bacterial primers targeting the V4 region of the bacteria 16S rRNA gene: 515F (5’- GTGACCTATGAACTCAGGAGTCGTGCCAGCMGCCGCGGTAA -3’ [43]) and 806R (5’- CTGAGACTTGCACATCGCAGCGGACTACHVGGGTWTCTAAT-3’ [44]) with overhang adapters (underlined). DNA sequencing libraries were prepared according to Aubrey *et al*. [45] No-template negative PCR controls (*n* = 3) were included to test for potential contamination. Triplicate PCRs were performed in 15 μl volumes comprised of 1 μl template DNA (1:20 dilution), 7.5 μl of 2 × MyTaq HS Mix polymerase (Meridian BioScience; USA), 0.45 μl of 10 μM forward and reverse primers, and 5.6 μl of nuclease-free water. Thermal cyclers were set to 1 cycle × 95°C for 3 min; 18 cycles × 95°C for 15 s, 55°C for 30 s and 72°C for 30 s; 1 cycle × 72°C for 7 min; hold at 4°C. Triplicate PCR products were then pooled; successful DNA extraction was confirmed by agarose gel electrophoresis.

A volume of 20 μl of each PCR product pool was purified by size selection using Nucleomag NGS clean-up and size select beads (Scientifix, Australia). The purified DNA was resuspended in 40 μl of nuclease-free water. Indexing PCRs were created by combining 10 μl of purified DNA with 10 μl 2 × MyTaq HS Mix polymerase (Meridian BioScience; USA) and 1 μl (5 μM) of forward and reverse indexing primers. Thermal cyclers were set to: 1 cycle × 95°C for 3 min; 24 cycles × 95°C for 15 s, 60°C for 30 s and 72°C for 30 s; 1 cycle × 72°C for 7 min; hold at 4°C. For a subset of randomly chosen samples, product size was confirmed by agarose gel electrophoresis. Sequencing libraries were created by pooling 5 μl from each reaction by plate (3 pools) and performing a final bead clean-up on 50 μl. Each library was checked for quality and quantity (2200 TapeStation, Agilent Technologies, Australia) to guide pool normalization, then sequenced on a single Illumina MiSeq run using v3 (2×300 bp) reagents at the Walter and Eliza Hall Institute, Melbourne, Australia.

### 2.6. Sequence data processing and statistical analysis

Sequence data analyses followed those of Dungan *et al.* [46]. Briefly, raw 16S rRNA gene sequences were imported into QIIME2 v2021.8 [47], where sequences were demultiplexed, primers removed (using cutadapt v2.6; [48]), then data filtered, denoized and chimera checked [using DADA 2; 49] to generate amplicon sequence variants (ASVs). Taxonomy for each ASV was assigned against a SILVA database (version 138) trained with a naïve Bayes classifier against the same V4-V5 region targeted for sequencing [50]. A phylogenetic tree was produced in QIIME2 by aligning ASVs using the PyNAST method [51] with mid-point rooting.

All data were analyzed in R (v4.2.1) [52]. Differences in male sperm viability were analyzed with a Wilcoxon test. Although two males had their microbiota assayed one day later, and following an additional fasting period (see above), we were unable to incorporate this into subsequent analyses due to the unbalanced sample size.

ASV, taxonomy, metadata and phylogenetic tree files were imported into R and combined into a phyloseq object [53]. Contaminant ASVs were identified and removed sequentially from the dataset according to their abundance in the extraction and PCR negative controls relative to the samples using the prevalence method in the R package decontam with *p* = 0.1 [54]. To determine the minimum sampling effort (rarefaction) sufficient to capture full diversity, rarefaction curves were generated by tissue type in R using ggplot2 [55].

Alpha diversity, which assesses microbial variation within a sample, was investigated to assess the impact of male reproductive and gut tissue type on the bacterial communities. We calculated the alpha-diversity on a rarefied dataset using the number of observed ASVs, Chao1 and Shannon’s and Simpson’s indices using the R package vegan [56]. Alpha diversity data were then analyzed using linear mixed-effects models, with male tissue type x sperm viability (low or high) and male weight as fixed effects, and male identity as a random effect, using the R package nlme [57]. Post hoc comparisons were performed using Tukey’s honest significant difference test in the R package emmeans [58]. Bar charts were created with ggplot2 [55] by agglomerating taxa at the phylum or genus level based on relative abundances using the R package phyloseq.

Beta-diversity, which assesses differences in microbial composition between samples, was evaluated using Bray–Curtis distance matrix and visualized using principal coordinates analysis (PCoA). The difference in community compositions among groups (sperm viability (low or high) and male tissue type) were calculated using adonis (a modified version of PERMANOVA; perm = 9999) in the vegan package in R [56] with a permutation test for multivariate dispersion to account for unequal dispersion between groups. Holm corrected post hoc pairwise comparisons were calculated using the function ‘pairwise.adonis’. Where the PERMANOVA test revealed that the community composition was significantly different (*p *< 0.05), linear discriminant analysis effect size (LEfSe) in the microbiomeMarker package [59] was used to identify bacteria taxa that were more dominant in each sperm viability cohort based on a *p *< 0.01 from the Wilcox test and LDA score>2.0 (we used a more conservative alpha level here to identify the most influential bacteria). The histogram and heatmaps were created to visualize the abundance and distribution of these taxa biomarkers by viability category.

## 3. Results

### 3.1. Sperm viability cohorts

In total, 97 males were assessed and ranked for the proportion of live sperm in their ejaculate (sperm viability) (median proportion of viable sperm (25th : 75th quartiles); 0.61 (0.53 : 0.69). Seven males did not produce a spermatophore on day 13 (and were tested for sperm viability on day 14 and microbiome on day 15). The 15 highest- and lowest-ranked males were then assigned to a ‘high’ and ‘low’ sperm viability cohort, respectively. Of these 30 males, only two were males that failed to produce a spermatophore on day 13, one each from the high and low sperm viability cohorts.

Our selection for male low and high sperm viability resulted in two cohorts of males that differed significantly in the sperm viability (low-viability cohort: 0.40 (0.37 : 0.46), high-viability cohort; 0.74 (0.73 : 0.81, *X* ^2^ = 21.80, *p* < 0.0001).

### 3.2. Metabarcoding data processing

Sequencing produced 2.3 M reads across the five tissue types for the 30 males (*n* = 149; one low sperm viability spermatophore was lost during processing), extraction blanks (*n* = 7) and PCR negative control samples (*n* = 3). Four samples (one gut, one seminal vesical and two spermatophore) had 0 reads and were removed during the QIIME2 processing. After merging, denoising and chimera filtering, 1.4 M reads remained. Decontam identified six putative contaminant ASVs from PCR amplification (1.04% of total reads), and six putative contaminant ASVs from DNA extraction (0.02% of total reads) (electronic supplementary material, table S1). Seven samples were removed from analysis as they had <400 reads (one gut, one seminal vesical, two accessory glands and two spermatophore samples). After all filtering steps, there were 1887 ASVs across the remaining cricket samples (*n* = 138).

### 3.3. Microbial diversity differs across male tissue types

**Figure 1.**
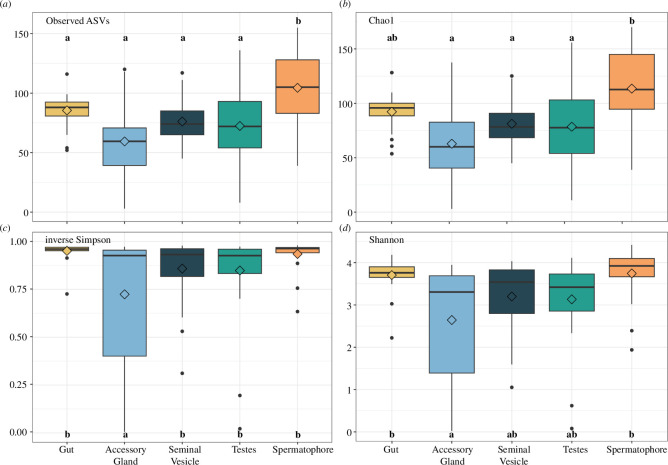
Alpha diversity indices (*a*) observed ASVs, (*b*) Chao1 index, (*c*) inverse Simpson’s index and (*d*) Shannon’s index by tissue type. Boxes cover the interquartile range (IQR) and the diamond inside the box denotes the median. Whiskers represent the lowest and highest values within 1.5 × IQR. Different lowercase letters signify significant differences in Tukey’s honest significant difference (HSD) post hoc tests.

Based on the rarefaction curve (electronic supplementary material, figure S1), samples were rarefied to 2133 reads, which removed 21 samples and 245 ASVs from the alpha diversity analysis. Alpha diversity indices indicated that male tissue type, but not sperm viability, significantly influenced the species richness of the microbiota present (figure 1; table 1; electronic supplementary material, figure S2). Microbial diversity in the spermatophore was significantly richer than all other tissue types for ASV and Chao1 indices (electronic supplementary material, table S2). For inverse Simpson’s index, which emphasizes species evenness and gives more weight to rare species, the accessory gland was significantly less diverse than the gut, seminal vesicle, testes and spermatophore. A similar trend is highlighted by the Shannon index, where both species richness and evenness are influenced by the abundance of different bacteria species. Here, the accessory gland was significantly less diverse than the gut and spermatophore only (electronic supplementary material, table S2, figure 1*d*).

**Table 1.** Statistics for alpha diversity metrics evaluated using a linear mixed-effects model. Significant comparisons (<0.05) are highlighted in bold. Pairwise comparisons for significant individual effects by tissue type are reported in electronic supplementary material, table S2.

alpha diversity metric	effect	statistic	***p***
observed ASVs	tissue type	*F*_4,79_ = 7.95	**<0.0001**
observed ASVs	sperm viability	*F*_1,28_ = 0.40	0.53
observed ASVs	tissue type * sperm viability	*F*_4,79_ = 1.02	0.40
chao1	tissue type	*F*_4,79_ = 7.84	**<0.0001**
chao1	sperm viability	*F*_1,28_ = 0.41	0.53
chao1	tissue type * sperm viability	*F*_4,79_ = 1.30	0.28
inverse Simpson	tissue type	*F*_4,79_ = 7.78	**<0.0001**
inverse Simpson	sperm viability	*F*_1,28_ = 0.23	0.64
inverse Simpson	tissue type * sperm viability	*F*_4,79_ = 0.06	0.99
Shannon	tissue type	*F*_4,79_ = 8.18	**<0.0001**
Shannon	sperm viability	*F*_1,28_ = 0.22	0.64
Shannon	tissue type * sperm viability	*F*_4,79_ = 0.03	1.00

**Figure 2.**
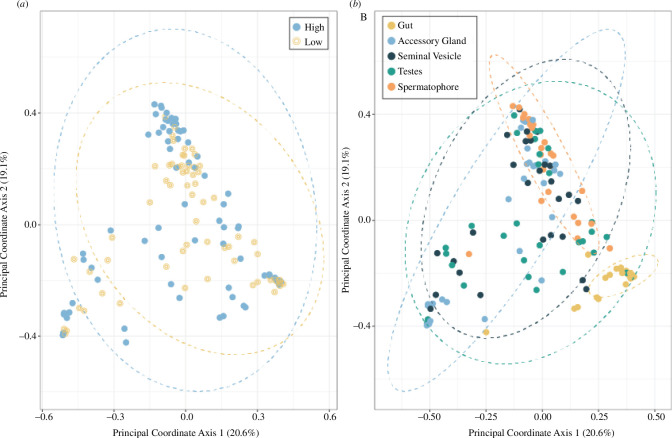
Principal coordinate analysis (PCoA) of reproductive tissue and gut samples (*n* = 138) using a Bray–Curtis distance matrix by sperm viability cohort (*a*) or tissue type (*b*). Axis 1 explains 20.6% of the variation while axis 2 explains 19.1%. Ellipses indicate 95% confidence intervals.

**Figure 3.**
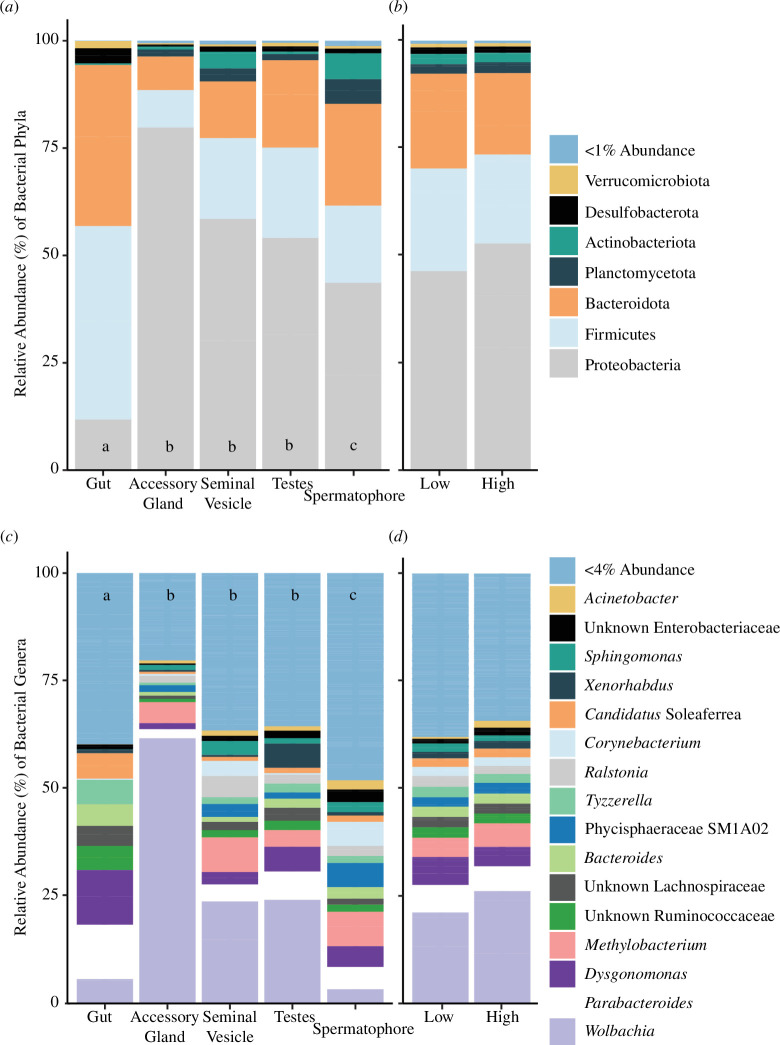
Relative abundance of bacterial phyla (*a,b*) and genera (*c,d*) present across the male tissue types (*a,c*) or by sperm viability (*b,d*). Phyla and genera whose relative abundance was less than 1 and 4%, respectively, across all tissue types and viability levels were pooled. Different lowercase letters for tissue type signify significant differences in Tukey’s honest significant difference (HSD) post hoc tests. There was no significant difference between the sperm viability cohorts.

PERMANOVA analysis revealed significant differences in community structure by sperm viability (*F*_1,137_ = 1.42, *p* = 0.0006; figure 2*a*) and tissue types (*F*_4,137_ = 7.06, *p *< 0.0001; figure 2*b*), but no significant interaction between tissue type and sperm viability (*F*_4137_ = 0.84, *p* = 0.59). Post hoc pairwise comparisons by tissue type, with Holm–Bonferroni corrected *P* values, reveal that the microbial communities from the gut were significantly different from each other tissue type (table 2), where the gut was dominated by phyla Firmicutes (genera *Tyzzerella, Candidatus soleaferrea* and Unknown Lachnospiraceae and Ruminococcaceae) and Bacteroidota (genera *Dysgonomonas* and *Bacteroides*) (figure 3). Spermatophore microbial community structure was also significantly different to all other tissue types (figure 3; table 2), with a notably reduced abundance of *Wolbachia* (electronic supplementary material, figure S3). We observed that *Wolbachia* in the accessory gland made up 73.6 and 53.0% of the total microbiome in the high and low sperm viability groups, respectively.

**Table 2.** Post hoc pairwise comparisons of microbial communities between tissue types. *Holm–Bonferroni adjusted *P* values. Significant comparisons (<0.05) are highlighted in bold.

pairs	sums of squares	*F*	*R* ^2^	*p*	***p*** adjusted *
spermatophore versus accessory gland	1.34	4.77	0.09	0.0001	**0.001**
spermatophore versus gut	3.99	19.31	0.27	0.0001	**0.001**
spermatophore versus seminal vesicle	0.78	2.90	0.05	0.0009	**0.004**
spermatophore versus testes	1.00	3.60	0.06	0.0003	**0.002**
accessory gland versus gut	4.28	17.13	0.23	0.0001	**0.001**
gut versus seminal vesicle	3.60	15.39	0.23	0.0001	**0.001**
gut versus testes	3.03	12.15	0.18	0.0001	**0.001**
accessory gland versus seminal vesicle	0.34	1.13	0.02	0.26	0.53
accessory gland versus testes	0.42	1.33	0.02	0.17	0.51
seminal vesicle versus testes	0.27	0.87	0.02	0.54	0.54

**Figure 4.**
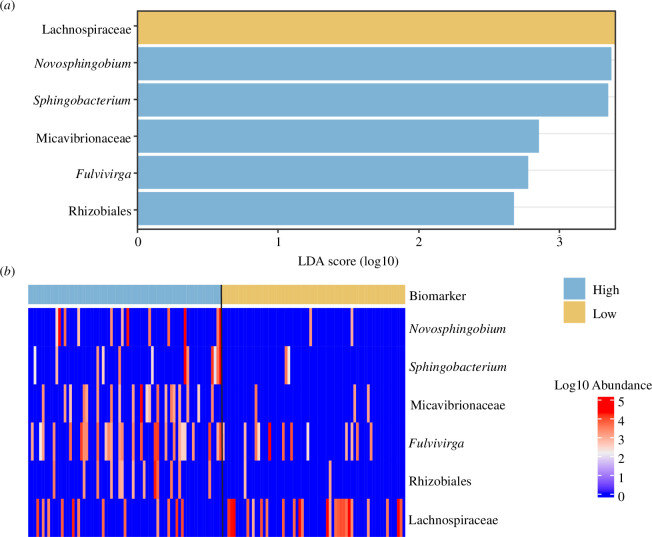
Histogram (*a*) shows the magnitude of the observed effect due to each biomarker using a linear discriminant analysis (LDA) score for high (blue) or low (yellow) sperm viability with LEfSe. The log10 normalized abundance of each of these biomarkers is shown in a heatmap (*b*) for all samples (each column is a sample).

LEfSe identified six taxonomic groups that were biomarkers of sperm viabilities (figure 4; table 3). Biomarkers for high sperm viability included the family Micavibrionaceae and genera *Novosphingobium*, *Sphingobacterium*, *Fulvivirga* and *Rhizobiales*, while only one family (Lachnospiraceae) was a biomarker for the low viability cohort. For the analysis of tissue type, LEfSe analysis identified 85 families or genera as biomarkers of tissue type (gut = 32, accessory gland = 12, seminal vesicle = 5, testes = 7, spermatophore = 29; electronic supplementary material, table S3). The families/genera with the greatest effect sizes for each tissue type were Phycisphaeraceae (SM1A02) and *Methylobacterium-Methylorubrum* (spermatophore), *Acinetobacter* and *Prevotella* (accessory gland), *Dysgonomonas* and *C. soleaferrea* (gut), Ralstonia and Bradyrhizobium (seminal vesicle) and *Pseudomonas* (testes).

**Table 3.** Taxa identified from LEfSe analysis of high and low sperm viability cohorts. Effect sizes are calculated using linear discriminant analysis scores. Here they reflect the strength and direction of the association between the identified bacteria and the sperm viability cohort.

biomarker	taxa	sperm viability cohort	effect size	***p***
1	*Novosphingobium*	high	3.37	0.006
2	*Sphingobacterium*	high	3.35	0.007
3	Micavibrionaceae	high	2.85	<0.001
4	*Fulvivirga*	high	2.78	0.007
5	NRL2 (Rhizobiales)	high	2.67	0.003
6	Lachnospiraceae	low	3.39	0.009

## 4. Discussion

Our study revealed a relationship between host–microbial diversity and natural variation in male fertility. Furthermore, we demonstrate significant variation in microbial diversity across the male reproductive tract, although this spatial variation did not directly contribute to differences in male sperm viability. Thus, we provide novel evidence of the localized and divergent nature of microbial communities among the gut and reproductive tract and highlight the potential for host microbes to shape normal variation in male fertility and influence post-copulatory sexual selection.

Primarily, evidence for the impacts of microbes on male fertility is sourced from cohort studies of infertile men or murine model systems [5,7,9,60]. Therefore, we know very little about how microbial variation affects fertility in non-clinical populations, and thus how host-associated microbes can affect post-copulatory sexual selection. Furthermore, studies have also typically focused on either gut or seminal fluid microbiomes [5,61], with relatively few studies directly exploring the relationship between host reproductive tissue microbiota and male fertility [7]. Six taxa were identified as being differentially represented in the high and low sperm viability cohort hosts, providing correlative evidence of their role in fertility. Higher relative abundances of Proteobacteria (*Novosphingobium*, Micavibrionaceae and *Rhizobiales*) and Bacteroidota (*Sphingobacterium* and *Fulvivirga*) were positively associated with sperm viability, and higher relative abundance of Firmicutes (*Lachnospiraceae*) with lower sperm viability. There are scant data regarding the functional role of the majority of these bacteria, and we note that these bacteria were not localized to one tissue type, which makes it difficult to assess their functional role in mediating fertility. Nevertheless, there is evidence for some genera and the role they may play in fertility in other host species. For example, in contrast to our findings, phylum Sphingobacteria is negatively correlated with sperm motility in humans [62]. Conversely, family *Lachnospiraceae* was associated with lower sperm viability in our study, which is consistent with a study that found a greater representation of this bacteria from this family in the rectum (as a proxy of the gut microbiome) of infertile men [60]. *Lachnospiraceae* are the main producers of short-chain fatty acids in the gut, which are associated in general with gut health, the reduction of inflammatory processes and the regulation of immunity [63]. Interestingly, a meta-analysis revealed that immunosuppressive corticosteroid treatments in men reduced antisperm antibody levels (as sperm is perceived as ‘non-self’ by the host [64]), and improved sperm motility count [65], and raises the possibility that the negative relationship between *Lachnospiraceae* and sperm viability may be mediated by immunological processes. Microbiome metabarcoding studies are necessarily correlative, so the directional relationship between postcopulatory sexual selection and host–microbial diversity remains unclear.

While our study found no direct association between the gut microbiome and male fertility, the gut is a major source of microbes in host systems, and there is increasing evidence of the potential for the gut microbiome to affect male fertility. Interestingly, indirect evidence of this relationship has been demonstrated in *T. oceanicus*, where separate studies demonstrate that high protein diets increase species evenness of the gut microbiome [40], but reduce sperm viability [66]. In murine model systems, dietary interventions to shift the gut microbial assemblages have provided insight into the relationship between the gut microbiome and male fertility. Mice fed a high-fat diet showed a decreased abundance of Bacteriodota and Verrucomicrobia and an increased abundance of Firmicutes and Proteobacteria in their gut microbiota, and a concomitant reduction in sperm concentration and motility [19]. Faecal transplants from alginate oligosaccharide-dosed mice to busulfan-treated mice increased the ‘beneficial’ bacteria Bacteroidales and Bifidobacteriales, and were associated with significant increases in both sperm concentration (twofold) and motility (20-fold) [20]. Similarly, alginate oligosaccharides can ‘rescue’ busulfan-disrupted spermatogenesis in mice along with an increase in ‘beneficial’ bacteria such as Bacteroidales and Lactobacillaceae and a decrease in ‘harmful’ bacteria such as Desulfovibrionaceae in the gut microbiota [67]. In humans, infertile men displayed significantly increased alpha-diversity in semen samples and distinct beta-diversity in both rectal (gut microbiome proxy) and seminal samples. The rectum of infertile men harboured decreased abundances of *Anaerococcus* and increased abundance of Lachnospiraceae, *Collinsella* and *Coprococcus* [60]. It should be noted that our study animals were fed a narrow, high-protein diet, which is likely to constrain gut microbial diversity [39], and thus, further studies should seek to reassess these relationships when males are permitted dietary choice. Nevertheless, evidence from other studies demonstrates the important link between gastrointestinal microbiomes and reproductive health and highlights the potential for dietary intervention to alter male fertility in a range of species [68].

Our results demonstrate significant variation in the diversity of microbiota across the reproductive tract. Some alpha diversity metrics indicate that the spermatophore has significantly greater species richness compared with the other reproductive tissues (but not the gut), while the accessory gland has a lower species richness compared with the gut and spermatophore. There are several potential explanations for these key differences. First, the spermatophore is likely to be a microbially species-rich tissue type due to the nature of its storage. Sexually mature male *T. oceanicus* produce a spermatophore, which sits in the male genital pouch and is exposed to the external environment, until they mate. Males will periodically extrude and replace old spermatophores to ensure their functional integrity. Nevertheless, the spermatophore is potentially exposed to bacteria in the external environment for a substantial period of time. Comparably, the reduced species richness of the accessory gland may also be due to its functional role in male reproduction. Male accessory glands in insects are involved in the synthesis and secretion of seminal fluid components, which have a variety of functions across taxa, including sperm activation, nutrient provisioning to females and manipulation of female reproductive behaviour, via fecundity-enhancement and/or post-mating receptivity inhibition (for a review, see [69]). However, seminal fluid also has anti-microbial properties. Microbes and sperm encounter each other frequently during reproduction [4], and these interactions are a strong selection pressure for the antimicrobial properties of the ejaculate [17,32,70–73]. Indeed, antimicrobial compounds have been identified in the seminal fluid of *T. oceanicus* [74]. It must be noted, however, that other tissues contribute to insect seminal fluid, including the seminal vesicles and testes [72], which did not demonstrate a comparable paucity of bacterial diversity.

Alternatively, the localized reduction in microbial variation in the accessory gland may be associated with the significantly greater relative abundance of *Wolbachia* in this tissue. *Wolbachia* has demonstrated negative impacts on host–microbial diversity, often becoming the dominant species once infected. Comparisons between naturally *Wolbachia*-free and *Wolbachia*-infected populations of small brown planthoppers, *Laodelphax striatellus,* show that infection severely decreases host–microbial diversity and abundance [75]. Similarly, in the cabbage root fly, *Delia radicum*, existing *Wolbachia* infection significantly decreases the microbial diversity of their host, changing their structure and composition by reducing abundance in some taxa but increasing others [76]. Experimental infection with *Wolbachia* (via injection) also altered host microbiota in the mosquito, *Aedes aegypti,* reducing the relative abundances, but not species diversity, of microbiota [77]. However, *Wolbachia’s* ability to suppress host–microbial diversity in a tissue-specific fashion, however, remains to be tested directly.

The impact of *Wolbachia* infection on male host fertility has been studied in various insects [78]. *Wolbachia*-infected *Drosophila simulans* males produce sperm cysts at a slower rate than uninfected males, which resulted in infected males producing approximately 40% fewer sperm cysts [79]. In addition, non-virgin *Wolbachia*-infected *D. simulans* males sire fewer progeny due to production of less competitive sperm [27]. In *Drosophila melanogaster, Wolbachia* can affect spermatid differentiation, chromosome deposition and sperm activity [80], leading to reduced fertility. Despite *Wolbachia’s* negative impact on male fertility in a range of species, our results suggest that the *Wolbachia* population was not associated with sperm viability in *T. oceanicus*. However, whether it affects other ejaculate quality metrics, such as sperm number, remains to be determined.

The most dominant phyla in the gut of *T. oceanicus* were Firmicutes (45%), Bacteroidota (38%) and Proteobacteria (12%). These phyla are prevalent in the guts of most insect orders, including other orthopterans [81–83], but also other taxonomically diverse groups, such as mammals [84]. The PCoA (figure 2*b*) showed the gut samples were clustered closely together, suggesting a specific set of microbes that have evolved to live in this environment and may reflect the narrow diet (commercially available cat chow) of this laboratory-adapted population. LeFSe analysis revealed 32 biomarkers of gut tissue type, with Dysgonomonadaceae, Ruminococcaceae, Lachnospiraceae, Bacteroidaceae, Anaerovoracaceae, Desulfovibrionaceae and Tannerellaceae accounting for the highest effect sizes (top 10) in this tissue type. In general, wild-caught organisms host more diverse gut microbial communities than laboratory populations [85,86]. Indeed, laboratory-reared *T. oceanicus* have a lower gut bacteria diversity compared with field-caught individuals, potentially due to a lower exposure to diverse environmental microbes (cleaner environment and food), but also because of the relatively homogenous diet [39]. Our characterization of the gut microbiota is highly comparable to a previous study on *T. oceanicus* also fed a commercially available cat chow (Firmicutes = 44%, Bacteroidota = 33%, Proteobacteria = 14%, Tenericutes = 3%, Fusobacteria = 3% and Deferribacteres = 3% (*n* = 6)) [39]. Furthermore, gut species evenness in *T. oceanicus* increases with higher protein consumption [40]. Crickets in our study were fed a diet of commercial cat chow, which has a high protein-to-carbohydrate ratio. In general, bacterial abundances are associated with dietary macronutrient ratios, for example, animals that consume high protein/low carbohydrate or animal-based diets have a lower abundance of polysaccharide-degrading Firmicutes and a higher abundance of Bacteroidota [87].

Finally, our experimental design was not able to determine if the microbial communities present in each of the reproductive tissues are stable or more transient assemblages. For some tissue types, such as the gut and spermatophore, there is likely to be a significant contribution from external sources, which also likely explains their greater species diversity. It is important to note that the males assayed in this study were virgins, a brief and temporary status. The potential for mating-related transfer of microbes, such as sexually transmitted infections, during mating is clear. Future studies should seek to examine whether changes in the post-mating microbial communities of the male reproductive tract may be associated with fertility. Understanding spatial and temporal variation in both reproductive and gut microbiomes and their relationship to sperm quality is critical for our understanding of male fertility. Nevertheless, our study provides novel evidence of the relationship between host microbial diversity and male postcopulatory sexual selection. Although the factors that mediate this relationship are yet to be determined, it provides impetus for further research into: the role of immune processes in these relationships and how microbial diversity is reflected in the trade-offs typically seen between an individual’s investment into pre- and post-copulatory sexual selection [88]. This may be particularly relevant in this model system, as diet causes opposing selection on pre-, and post-copulatory sexual selection, and also alters gut microbiota.

## Data Availability

Data and relevant code for this research work are stored in GitHub [89] and have been archived within the Zenodo repository [90]. Supplementary material is available online [91].
